# Real-life data on pasireotide in monotherapy or combined in active Cushing’s disease

**DOI:** 10.3389/fendo.2025.1695342

**Published:** 2025-11-13

**Authors:** Alessandro Mondin, Filippo Ceccato, Carla Scaroni, Luca Denaro, Renzo Manara, Umberto Maria Robertazzo, Mattia Barbot

**Affiliations:** 1 Department of Medicine (DIMED), University of Padova, Padova, Italy; 2 Endocrinology Unit, University Hospital of Padova, Padova, Italy; 3 Academic Neurosurgery, Department of Neurosciences, University of Padova, Padova, Italy; 4 Department of Neuroscience (DNS), University of Padova, Padova, Italy; 5 Neuroradiology Unit, University-Hospital of Padova, Padova, Italy

**Keywords:** Cushing’s disease, somatostatin analogue, pasireotide, combination treatment, metyrapone, cabergoline

## Abstract

**Introduction:**

In the last few years, the use of medical treatment in Cushing’s disease (CD) has increased thanks to the availability of new molecules. Nevertheless, real-life data on combination treatments are still lacking.

**Methods:**

This is a retrospective monocentric study evaluating the real-life efficacy and safety of pasireotide alone or when combined with cabergoline or metyrapone.

**Results:**

A total of 18 patients (94% women; median age, 45 years) with active CD received pasireotide (median, 8 months), followed by a combination treatment (median, 22 months) with either cabergoline (2/9) or metyrapone (7/9) for half of the patients. Pasireotide alone significantly reduced urinary free cortisol (UFC) and late-night salivary cortisol (LNSC) (*p* < 0.01), achieving normal values in 59% and 38% of cases, respectively. The second cortisol-lowering agent tended to further reduce UFC (overall normalization, 67%) but had little effect on LNSC. Pasireotide led to significant hyperglycemia in 61% of cases, while the add-on drug was well-tolerated. Comorbidities were analyzed accounting for the individual cardiovascular risk and for changes in concomitant treatments. Half of the patients showed improved pressure profile. Cholesterol levels tended to decrease, and a significant weight loss was observed (>5% in 47% of cases). Add-on treatment with dose reduction of pasireotide allowed better glycemic control in one of two cases.

**Discussion:**

Our experience confirms the efficacy of pasireotide on the UFC, especially in combined regimens, but also the difficulty of restoring circadian rhythm in CD. This is the first study to report metyrapone add-on to pasireotide, but larger studies are needed to further investigate this association. Pasireotide surely worsens glucose homeostasis, but its positive effects, alone or combined, on blood pressure, lipid profile, and body weight justify its use under careful hyperglycemia management.

## Introduction

Surgery is the first-line treatment for Cushing’s disease (CD) when feasible (1). The use of medical treatment has gained interest in recent years, particularly with the availability of new compounds (2–4). Medical therapy can be applied in several settings such as preoperatively, when surgery is contraindicated or refused, in persistent or relapsed cases after surgery, and while waiting for the effect of radiotherapy. The drug choice should be individualized, based on the severity of hypercortisolism, gender, clinical features, radiological features of the adenoma, comorbidities, and local availability (1). Except for glucocorticoid receptor antagonists, treatment monitoring should consider hormonal indexes on top of clinical evaluation. The most commonly used parameter is urinary free cortisol (UFC), often combined with morning serum cortisol to identify adrenal insufficiency. In recent years, the restoration of circadian rhythm has also emerged as a further marker of effective disease control (5, 6). In case of inadequate control, using a combination strategy may offer the advantage of better clinical and biochemical control as well as the possibility of reducing the dose of each drug, thus limiting its adverse effects.

Pasireotide is a somatostatin receptor multiligand active also on isoform 5, which underlies its effectiveness in CD as opposed to first-generation analogues (7). Although burdened by the risk of iatrogenic diabetes, pasireotide proved effective in controlling cortisol excess (2, 8) and may induce tumor shrinkage in a subset of patients by directly targeting the pituitary adenoma (9). To date, its use in combination with cabergoline and ketoconazole was reported in dedicated clinical trials (10, 11), whereas real-world data on combined strategies remain limited.

We aimed to report our experience with pasireotide, used either as monotherapy or in combination, for the treatment of active CD.

## Methods

We analyzed a retrospective cohort of patients with CD followed at our center who received on-label treatment with subcutaneous pasireotide between 2012 and 2024 outside multicenter clinical trial participation in order to evaluate the real-life experience with this drug. Data on a subset of the included patients have already been reported in previous studies on coagulative profile and iatrogenic diabetes carried out at our center (12–14). Eligible cases presented an active disease, either *de novo* or persisting/relapsing after other treatments (such as surgery and/or radiation). CD diagnosis was established based on coherent imaging of the sella and dynamic tests (corticotropin test, high-dose dexamethasone suppression test, and/or desmopressin test) and in some cases with a bilateral inferior petrous sinus sampling. For patients submitted to surgery, the diagnosis was confirmed by histological findings and/or persistent postoperative hypocortisolism (1, 15). Patients were excluded in case of lacking data or inadequate adherence to treatments and/or follow-up. Pasireotide was administered as per clinical practice with safety evaluations at 2 and 4 weeks and then every 2–3 months during the first year. Informed consent for the study was collected (PITACORA, protocol number AOP3318, Ethic Committee registration 5938-AO-24).

We assessed disease severity at baseline and its control during follow-up using UFC and late-night salivary cortisol (LNSC). The former was assessed via the mass spectrometry method (Agilent Technologies, Palo Alto, USA), while the latter was initially assessed with a radioimmunoassay (Radim, Rome, Italy) and then via mass spectrometry since 2014 (Agilent Technologies, Palo Alto, USA). ACTH was evaluated with an immunometric assay. We considered the most recent untreated values of ACTH and cortisol in the year prior to pasireotide start as baseline. The severity of hypercortisolism was categorized according to UFC levels as mild [<2-fold the upper limit of normal (ULN)], moderate (from 2- to 5-fold the ULN), or severe (>5-fold the ULN). UFC and LNSC values were evaluated as the mean of at least two values in order to account for their variability (16, 17). In patients with persistently abnormal circadian rhythm despite normal UFC, a treatment intensification aiming to normalize LNSC was pursued whenever deemed feasible and safe.

Pituitary magnetic resonance imaging (MRI) was performed before pasireotide initiation and then repeated during follow-up as per routine clinical practice using a 1.5- to 3-T field scanner.

We assessed metabolic and cardiovascular CD comorbidities and their treatments. Arterial hypertension was defined based on systolic and diastolic blood pressure (SBP ≥ 140 mmHg and/or DBP ≥ 90 mmHg) measurements during ambulatory visits (18) or in case of ongoing antihypertensive drugs; as per clinical practice, elevated values were confirmed after a resting period. Diabetes and impaired fasting glucose were defined according to the most recent guidelines (19) based on glycemia and glycosylated hemoglobin (HbA1c). No patient of our cohort had a recent oral glucose tolerance test prior to pasireotide start, although some received mixed meal test as a part of a different study protocol (14). We also registered the non-insulin antidiabetic agents used and the need for insulin treatment. Dyslipidemia was defined based on elevated triglycerides (>150 mg/dL), reduced high-density lipoprotein (HDL, below 50 mg/dL in female and 40 mg/dL in male patients), elevated total cholesterol (>200 mg/dL), calculated low-density lipoprotein (LDLc) cholesterol above the cardiovascular (CV) risk-based target, or in case of ongoing lipid-lowering treatment (20). Regarding CV risk, according to current guidelines, all patients with active disease were considered at high risk; we identified a very high CV risk based on the general population criteria from the guidelines (e.g., documented vascular disease or severe renal impairment) (1, 20). Overweight and obesity were defined and assessed via weight and body mass index (BMI) (21); waist circumference (WC) and the use of anorectic drugs were recorded. We assessed the clinical impact of pasireotide (alone or in combination with another drug) on comorbidities. Specific criteria to define the effect on pressure profile are reported in **Supplementary Table 1**.

### Statistical analysis

Categorical variables were reported as counts or percentages, and quantitative variables were reported as median and interquartile ranges (IQRs). The comparisons between independent groups were performed with non-parametric tests, namely, a Mann–Whitney sum rank test for quantitative variables and a chi-square test for categorical ones. Significance threshold was set at *p*-value <0.05. Statistical analyses were performed with R [R Core Team (2022), R: A language and environment for statistical computing, R Foundation for Statistical Computing, Vienna, Austria; https://www.r-project.org/] and R studio [Posit team (2024), RStudio: Integrated Development Environment for R, Posit Software, PBC, Boston, MA; http://www.posit.co/].

## Results

Eighteen patients were included in this study, mainly women with a median age at pasireotide treatment start of 45 [34; 53] years. Three *de novo* CD cases received pasireotide in the preoperative period, while all other cases had undergone at least one pituitary surgery. Five patients were irradiated 1, 15, 15, 17, and 30 years, respectively, prior to pasireotide start. Most received other medical treatments prior to pasireotide (**Table 1**). Three out of four macroadenomas showed cavernous sinus invasion. Histology was available in 13/17 operated cases, with 12 cases showing ACTH staining at immunochemistry; in 2 cases, adenomas showed high proliferative activity (Mib-1 >3%).

**Table 1:** Baseline features of our cohort prior to pasireotide start.

Baseline features	Number (%)
Female	17/18 (94%)
Arterial hypertension	14/18 (78%)
Impaired glucose homeostasis ✓ Impaired fasting glucose ✓ Diabetes mellitus	3/18 (17%) 0/3 (0%) 3/3 (100%)
Dyslipidemia ✓ High triglycerides ✓ Low HDL ✓ Elevated total cholesterol ✓ LDLc above CV risk-specific target*	16/18 (89%) 5/18 (28%) 2/18 (11%) 10/18 (56%) 16/18 (89%)
Weight excess ✓ Overweight ✓ Obesity	9/17 (53%) 6/9 (67%) 3/9 (33%)
Prior surgery ✓ 1st surgery ✓ 2nd surgery	15/18 (83%) 11/15 (73%) 4/15 (27%)
Prior pituitary irradiation	5/18 (28%)
Previous cortisol-lowering treatments ✓ Ketoconazole ✓ Metyrapone ✓ Cabergoline ✓ Pasireotide**	14/18 (78%) 9/14 (64%) 3/14 (21%) 6/14 (43%) 4/14 (29%)
Hypercortisolism severity*** ✓ Mild ✓ Moderate ✓ Severe	16/18 (89%) 7/16 (44%) 8/16 (50%) 1/16 (6%)
Magnetic resonance imaging ✓ Negative ✓ Microadenoma ✓ Macroadenoma	18/18 (100%) 7/18 (39%) 7/18 (39%) 4/18 (22%)

*Two patients presented very high CV risk (target LDLc <55 mg/dL) due to prior ischemic stroke and diabetes with multiple risk factors, respectively; the remaining patients were considered at high CV risk based on active CD (target LDLc <70 mg/dL). **Some patients already received a previous cycle with pasireotide s.c. bid or the long-acting release formulation i.m. during multicenter clinical trials. ***For two patients, untreated values in the year before pasireotide start were not available. CD, Cushing’s disease; HDL, high-density lipoprotein; LDLc, calculated low-density lipoprotein; CV, cardiovascular.

All patients initially received pasireotide as monotherapy. Combination therapy was subsequently introduced in nine patients (50%), using cabergoline (2/9, 22%) or metyrapone (7/9, 78%). The main reason for the add-on therapy was an inadequate control of hypercortisolism (7/9, 78%): five cases still presented abnormal UFC on pasireotide while two patients had UFC < ULN but impaired circadian rhythm. In one patient, the combination was prompted by concerns over tumor growth, while in another case, it was used to reduce pasireotide dosage in order to improve tolerability. At the start of pasireotide treatment, most of the patients harbored a microadenoma (14/18, 78%) (**Table 1**). Recent (i.e., within a year prior to pasireotide start) untreated values of UFC and LNSC were available for 16 and 14 cases, respectively, with median values of 2.08 [1.63; 3.39] and 2.15 [1.67; 4.08] times the ULN, respectively. Two patients switched from ketoconazole to pasireotide without a wash-out evaluation of hypercortisolism due to persistent impaired circadian rhythm despite normal UFC. In two other cases, only UFC prior to pasireotide therapy was evaluated; both cases showed impaired LNSC while on the prior treatment (ketoconazole and ketoconazole plus cabergoline, respectively).

### Efficacy of pasireotide monotherapy

Pasireotide was administered alone for a median treatment duration of 8 months [4; 23]. Dosages are reported in **Table 2**. Regarding hypercortisolism control, pasireotide monotherapy led to a significant reduction in median cortisol levels compared to the last available untreated values (**Table 3**). UFC decreased 39% [15%; 88%] and LNSC decreased 23% [2%; 57%]. A trend toward reduced ACTH levels was also observed (**Table 3**). Pasireotide alone led to UFC normalization in 8/16 cases, and the UFC remained normal in the two patients switched from ketoconazole (normal overall 10/18, 59%); half of the patients experienced a reduction greater than 50% (8/16). Circadian rhythm was impaired in all cases prior to pasireotide; 14 were evaluated off-treatment and 4 were evaluated during prior medical treatment (ketoconazole, *n* = 3; ketoconazole plus cabergoline, *n* = 1). LNSC data were not obtained in two patients while on pasireotide treatment. Among patients with available follow-up data, LNSC normalization was obtained in 6/16 (38%). A reduction of at least 50% was found in one-third of patients (4/12). No significant differences in treatment response were observed based on adenoma size (microadenoma vs. macroadenoma) or disease severity at baseline (mild vs. moderate/severe).

**Table 2:** Maximum and minimum dosages of the drugs administered to each patient.

Patient	Pasireotide		Cabergoline		Metyrapone*	
	Minimum	Maximum	Minimum	Maximum	Minimum	Maximum
1	300 μg bid	600 μg bid				
2	600 μg bid	600 μg bid				
3	600 μg bid	600 μg bid				
4	300 μg bid	600 μg bid				
5	600 μg bid	600 μg bid				
6	300 μg bid	600 μg bid				
7	300 μg die	600 μg bid				
8	600 μg bid	600 μg bid				
9	600 μg bid	900 μg bid				
10	300 μg bid	600 μg bid	1 mg week	2 mg week		
11	600 μg bid	900 μg bid	1 mg week	3 mg week		
12	300 μg bid	600 μg bid			500 mg die (0–0–2)	1000 mg die (1–1–2)
13	600 μg bid	600 μg bid			250 mg die (0–0–1)	250 mg die (0–0–1)
14	600 μg bid	900 μg bid			500 mg die (0–1–1)	500 mg die (0–1–1)
15	600 μg bid	600 μg bid			250 mg die (0–0–1)	1000 mg die (1–1–2)
16	300 μg bid	600 μg bid			500 mg die (0–1–1)	1250 mg die (1–2–2)
17	600 μg bid	600 μg bid			250 mg die (0–0–1)	1000 mg die (1–1–2)
18	600 μg bid	600 μg bid			250 mg die (0–0–1)	250 mg die (0–0–1)

*The daily distribution of the number of tablets (250 mg each) administered is reported (morning–midday–late evening).

**Table 3:** ACTH and cortisol values at baseline and following pasireotide alone or in combination.

	All patients				Combined treatment group			
	*N*	Baseline (untreated)	Pasireotide monotherapy	*P*	*N*	Pasireotide monotherapy	Combination treatment*	*P*
UFC (ULN-fold)	16	2.08 [1.63; 3.39]	0.97 [0.37; 1.44]	**<0.01**	9	1.08 [0.86; 1.36]	0.76 [0.42; 1.08]	0.26
LNSC (ULN-fold)	12	2.18 [1.79; 3.83]	1.84 [1.29; 2.07]	**<0.01**	8	2.05 [1.62; 2.37]	2.06 [1.29; 2.10]	0.55
ACTH (ng/L)	15	56.0 [26.5; 67.3]	32.0 [21.0; 49.5]	*0.07*	8	49.5 [31.8; 61.3]	46.5 [36.3; 79]	0.36

*Similar results were obtained considering only metyrapone as the add-on treatment. UFC, urinary free cortisol; LNSC, late-night salivary cortisol; ACTH, adrenocorticotropic hormone; N, number. Bold values indicate statistically significant differences.

### Efficacy of combination treatment

The add-on was performed after a median of 5 [3; 10] months of pasireotide therapy and was carried out for a median duration of 22 [2; 54.5] months with dosages detailed in **Table 2**. Compared to the last follow-up on pasireotide monotherapy, the addition of a second compound resulted in a further, though not statistically significant, reduction in UFC levels (median decrease, 33% [−15%; 63%]) (**Table 3**), with two additional patients reaching UFC normalization (from four to six total cases). The effect on circadian rhythm was poor (median decrease, 6% [−20%; 44%]) (**Table 3**). Among patients receiving metyrapone as add-on treatment, a slightly greater improvement in LNSC was observed (median decrease, 14% [−45%; 61%]). However, no additional patient achieved LNSC normalization with combination treatment (overall 6/16, 38%). On combined treatment, ACTH did not change significantly compared to pasireotide monotherapy. Cabergoline add-on had little effect on ACTH, while metyrapone add-on could lead to either its increase (*n* = 4) or decrease (*n* = 2); ACTH changes in these two groups did not significantly differ (*p* = 0.50). In three cases, the add-on was accompanied by a reduction of pasireotide dosage (from 600 μg BID to 300 μg BID) due to poor tolerance (*n* = 1) or hyperglycemia (*n* = 2). The patient presenting poor tolerance did not achieve hypercortisolism control despite the metyrapone add-on, and thus, pasireotide dose was increased again to 600 μg BID this time without issues. The other two cases presented UFC reduction >50% with cabergoline and metyrapone add-on, respectively.

### Safety

**Figure 1:**
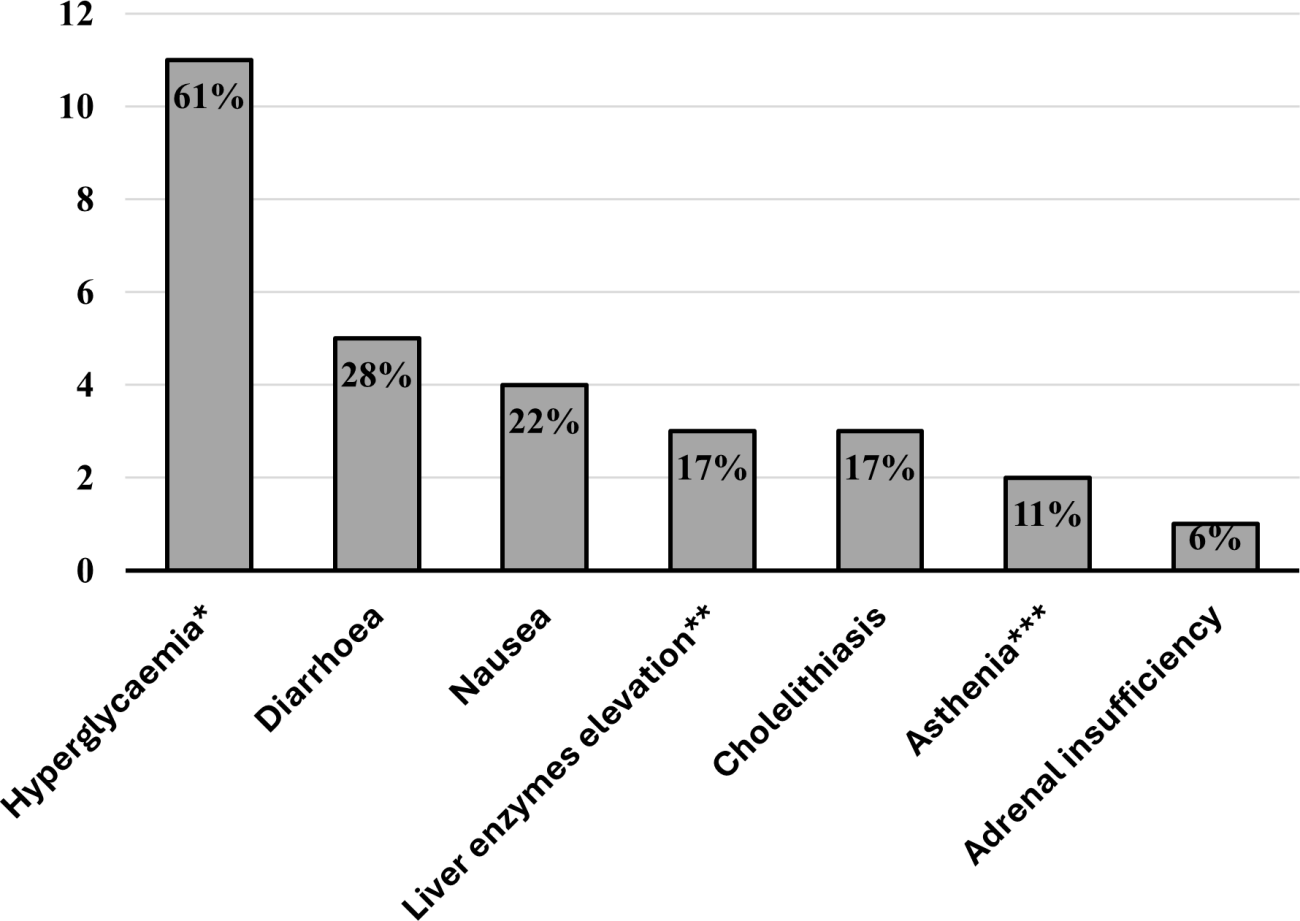
Bar chart displaying the percentages of patients experiencing pasireotide-related adverse events. *All cases were asymptomatic—we included new-onset diabetes or worsening of pre-existing diabetes. **<2-fold the upper limit of normal. ***Normal blood pressure values, ions, and serum morning cortisol.

Patients reported various adverse events during pasireotide treatment (**Figure 1**), with hyperglycemia occurring in more than 60% of cases. Gastrointestinal manifestations were mild and self-limiting in all cases. No serious adverse event and no pathological QT elongation was registered. Cabergoline was well tolerated, with no reported adverse events. In contrast, metyrapone led to mild and self-limiting nausea in two cases.

Most patients (15/18) discontinued pasireotide, with reasons detailed in **Supplementary Figure 1**. Among patients with at least 1 year of follow-up (*n* = 11), pituitary imaging remained stable in nine cases (eight micro- and one macro-adenoma). One patient exhibited tumor enlargement (from micro-to macro-adenoma) requiring reoperation. Conversely, one previously irradiated patient experienced shrinkage from macro- to micro-adenoma.

### Comorbidities

We evaluated the effect of pasireotide (alone or in combination with another drug) on comorbidities in the whole cohort (**Table 4**).

**Table 4:** Changes from baseline following pasireotide (alone or combined) treatment.

Parameter	Premises	Patients	Baseline	Last visit	*P*
SBP (mmHg)	Unchanged number/dosage of antihypertensives	7	130.0 [127.5; 132.5]	120.0 [110; 130]	0.33
DBP (mmHg)	Unchanged number/dosage of antihypertensives	7	80.0 [77.5; 82.5]	80.0 [77.5; 82.5]	0.59
Glycemia (mg/dL)	No glucose-lowering drugs	7	77.0 [72.0; 88.5]	85.0 [78.0; 88.5]	0.34
HbA1c (mmol/mol)	No glucose-lowering drugs	7	31.0 [27.0; 33.0]	39.0 [31.0; 40.0]	**0.02**
Glycemia (mg/dL)	Start/increase in glucose-lowering drugs/insulin use	11	81.0 [69.0; 86.0]	133.0 [121.5; 164.5]	**<0.01**
HbA1c (mmol/mol)	Start/increase in glucose-lowering drugs/insulin use	11	39.0 [36.5; 40.0]	54.0 [53; 62.5]	**<0.01**
Cholesterol (mg/dL)	Unchanged number/dosage of lipid-lowering drugs	12	208.5 [188.3; 227.3]	198.0 [178.8, 204.3]	*0.08*
HDL (mg/dL)	Unchanged number/dosage of lipid-lowering drugs	12	60.5 [51.8; 65.0]	61.5 [49.8; 69.0]	1.00
Triglycerides (mg/dL)	Unchanged number/dosage of lipid-lowering drugs	12	77.0 [57.5; 137.0]	85.5 [48.5; 147.8]	0.66
LDLc (mg/dL)	Unchanged number/dosage of lipid-lowering drugs	12	123.9 [106.1; 149.9]	110.7 [87.4; 117.5]	*0.09*
BMI (kg/m^2^)	No concomitant use of anorectic drugs	15	24.3 [22.1; 27.8]	23.4 [20.7; 26.5]	**0.02**
WC (cm)	No concomitant use of anorectic drugs	13	91.0 [83.0; 106]	85.0 [81.0; 99.0]	0.11

SBP, systolic blood pressure; BDP, diastolic blood pressure; HbA1c, glycosylated hemoglobin; HDL, high-density lipoproteins; LDLc, calculated low-density lipoproteins; BMI, body mass index; WC, waist circumference. Bold values indicate statistically significant differences.

Arterial hypertension was prevalent at baseline (**Table 1**) and untreated in two cases; most patients received combined antihypertensive treatments with mineralocorticoid receptor antagonists being the most commonly used drugs (*n* = 5), although in some cases, their main indication was to reduce androgenic symptoms, followed by calcium channel blockers and angiotensin-converting enzyme inhibitors (*n* = 4 each). One patient discontinued antihypertensive treatment during pasireotide treatment. BP values and treatments were assessed after pasireotide (alone or combined) treatment and compared to baseline according to the criteria reported in **Supplementary Table 1**. Overall, nine patients presented an improvement of their pressure profile while only three patients showed a worsened profile (none of whom received metyrapone); the remaining five patients showed a stable course. Among patients treated for at least 1 year, we found that half of the patients (*n* = 5) exhibited an improved BP profile with none showing deterioration (**Supplementary Table 1**). Patients who did not modify antihypertensive treatment did not show significant changes in their BP (**Table 4**).

At baseline, three patients had diabetes (two on metformin and one on diet treatment), while all others presented with normal glucose homeostasis based on fasting glycemia and HbA1c. During follow-up, 11 patients required initiation or intensification of anti-diabetic treatment (only one patient with a pre-existent diabetes required insulin), but glycemic profile remained worse compared to baseline. Patients not receiving antidiabetic treatment during the pasireotide (alone or in combination) course also showed a worsening HbA1c, although it remained within normal range in all cases (**Table 4**).

Considering the criterion of elevated LDLc for a high CV risk population, most of our patients were dyslipidemic (16/18, 89%), 4 of whom despite ongoing lipid-lowering drugs at baseline. Five of these patients also presented with hypertriglyceridemia and two had a reduced HDL value. At the last available follow-up, treatment intensification was registered in six patients (additional drug or increase in dosage/intensity of the statin used), three of whom (50%) reaching desirable LDLc levels. Nevertheless, 14 patients still had LDLc >70 mg/dL at the last visit, and the prevalence of hypertriglyceridemia remained unchanged (3/18, 17%). Considering only patients without treatment intensification, there was a trend towards reduced levels of total and LDLc cholesterol (**Table 4**).

At baseline, 8/17 patients were overweight and 2 were obese. Two patients receiving glucagon-like peptide 1 analogs (aGLP1) for iatrogenic diabetes were excluded from analyses regarding weight and WC. At the last visit, there was a significant reduction in BMI, while a decrease in WC did not reach significance (**Table 4**). Out of 15 patients, 7 (47%) experienced a weight decrease >5%.

Comparing combined treatment to pasireotide monotherapy (*n* = 9), no significant difference in glycemia and HbA1c was detected, with glucose-lowering treatments unchanged, intensified, or reduced in three cases each (33% each). Except for two patients who had increased antihypertensive treatment with coherent BP reduction, SPB and DBP values remained stable. Three patients started/increased lipid-lowering treatment, while five patients maintained the same therapy and showed a trend of LDLc reduction (108.6 mg/dL vs. 88.6 mg/dL, *p* = 0.06). Excluding the two patients receiving anorectic drugs, a trend of reduction in BMI (22.9 vs. 21.1 kg/m^2^, *p* = 0.09) and WC (95 vs. 90 cm, *p* = 0.09) also emerged with the addition of a second compound.

As previously mentioned, two patients received add-on treatment but reduced pasireotide dosage due to hyperglycemia. The patient receiving metyrapone obtained a 7-mmol/mol reduction of HbA1c without glucose-lowering treatment modifications; conversely, the patient receiving cabergoline required an intensification of glucose-lowering treatment to improve glycemic control.

## Discussion

Pasireotide use in CD is supported by large clinical trials and real-life reports, but data on its use in combination with other drugs are less abundant.

In our cohort, pasireotide proved effective in lowering cortisol levels, allowing normalization of UFC and LNSC in 59% and 38% of cases, respectively, with limited risk of overtreatment (only one case of iatrogenic hypocortisolism). In a previous Italian multicenter study, pasireotide led to UFC normalization or a >50% decrease from baseline in 93.8% and 100.0% of patients after 6 and 12 months, respectively (22).

Few studies on combination treatment in CD are available to date (**Table 5**) (10, 11, 23–29), often as a part of clinical trials. In contrast, our study focused on real-world data to describe how this approach is implemented in routine clinical practice.

**Table 5:** Studies on combination treatment in Cushing’s syndrome (CS).

Article, year	Drugs*	Population	Number	Outcomes*
Vilar, 2010 (23)	Cabergoline, Ketoconazole	CD	9	UFC normalization in 67% of cases
Feelders, 2010 (10)	Pasireotide, Cabergoline, Ketoconazole	CD	12	UFC normalization in 83% of cases
Kamenicky, 2011 (24)	Mitotane, Metyrapone, Ketoconazole	Severe CS (CD/EAS)	11	UFC normalization in 64% of cases**
Valassi, 2012 (25)	Metyrapone, Ketoconazole	CS	22	UFC normalization in 46% of cases
Barbot, 2014 (26)	Cabergoline, Ketoconazole	CD	14	UFC normalization in 79% of cases.
Corcuff, 2015 (27)	Metyrapone, Ketoconazole, Mitotane	Severe CS (EAS/ACC)	22	UFC normalization in 77% of cases
Dormoy, 2023 (28)	Osilodrostat, Ketoconazole, Metyrapone, Cabergoline	EAS	9	UFC normalization in 78% of cases***
Feelders, 2023 (11)	Pasireotide, Cabergoline	CD	42	UFC normalization in 41% of cases
Paes, 2025 (29)	Ketoconazole, Octreotide	CD	11	n.a.****

*Reported outcomes refer to patients receiving multiple agents (at least two of the listed) to achieve disease control.

**Based on **Figure 2** of the original article.

***Based on nadir urinary free cortisol (UFC) in **Table 2** and patient’s specific ULN from **Table 1**.

****Octreotide was added in biochemically controlled patients, with two cases presenting efficacy escape and requiring octreotide dosage increase to restore UFC values. CD, Cushing’s disease; EAS, ectopic ACTH secretion; ACC, adrenocortical cancer.

To the best of our knowledge, this is the first report describing the association of pasireotide and metyrapone. The rationale for this approach lies in using a combination that directly inhibits both levels—the pituitary ACTH secretion and the adrenal cortisol synthesis—thereby improving the hormone control potentially with lower drug doses and therefore less side effects (30). Notably, unlike other steroidogenesis inhibitors, metyrapone has not been associated with significant QT elongation (31), making it a more suitable option in combination with pasireotide. Moreover, some studies reported concern for tumor growth during treatment with steroidogenesis inhibitors (32), which may be addressed by the association with pasireotide. In our experience, metyrapone add-on allowed dose reduction of pasireotide, particularly in cases of secondary hyperglycemia. Another consideration is that adding metyrapone further decreased UFC, but it did not result in a parallel decrease of LNSC, confirming the challenge of restoring circadian rhythm with medical therapy in CD. Is not only the disruption of this rhythm a diagnostic hallmark of endogenous hypercortisolism (33), but also a potentially efficacious treatment target. Uncontrolled LNSC seems related to residual morbidity in otherwise well-controlled (i.e., normal UFC) patients, as recent studies showed that restoring rhythm could lead to further clinical benefits (6, 26, 34). Similarly, mimicking correct circadian rhythm when administering glucocorticoid treatment in adrenal insufficiency proved beneficial on clinical outcomes (6). Despite the chrono-pharmacologic approach used in our center [i.e., giving the main dose of short-acting steroidogenesis inhibitors in the second half of the day (35)], LNSC remained impaired: dedicated clinical trials on this topic would be beneficial to identify the best strategy to target this parameter.

Regarding cabergoline add-on, its effectiveness was already reported in a dedicated clinical trial (11), with the rationale of targeting the corticotroph adenoma with both drugs. Indeed, unlike steroidogenesis inhibitors, both these drugs target the adenoma with two different pathways: the somatostatin receptor (especially isoform 5) by pasireotide and the dopamine receptor 2 by cabergoline. Both drugs showed *in vitro*/*in vivo* potential antitumoral effects (36–41) and had induced tumor shrinkage of corticotroph adenomas in previous reports (9).

In the overall cohort, normalization of UFC and LNSC was reached in 67% and 38% of cases at the last follow-up, respectively.

The safety profile of pasireotide in our study was consistent with that reported in clinical trials, with hyperglycemia being the prominent adverse event. The management of iatrogenic diabetes in this setting was previously addressed in a dedicated phase IV study (42) that recommended the use of incretin-based drugs along with metformin, considering the underlying pathophysiology of pasireotide-related diabetes (14, 43), with insulin reserved as a rescue measure. In our cohort, only one patient required insulin treatment, whereas others received metformin alone or combined with other antidiabetic agents (three with dipeptidyl peptidase; two with GLP1, and one with a sodium glucose transporter 2 inhibitor). In line with the trial results, not all our patients required anti-diabetic treatments, despite the impaired glucose homeostasis. Both cabergoline and metyrapone were well tolerated in our cohort.

In our cohort, we registered a high discontinuation rate (**Supplementary Figure 1**); as four cases were subjected to pituitary surgery, drug-related withdrawal amounted to 61% (11/18) of the patients. Six patients requested a therapy switch as they were unwilling to continue twice-daily subcutaneous injections (two of them despite normalization of UFC values on their regimens). This compliance issue is relevant as one-monthly i.m. formulation of pasireotide is not currently available for CD in Italy, despite available literature supporting it (44). This is particularly relevant as a highly effective alternative with a favorable administration regimen and safety profile has emerged. Osilodrostat administered orally twice daily was reported to normalize UFC in up to 90% of cases (45), although few real-life data are available. The association of osilodrostat to pasireotide could achieve interesting results, but safety issues such as QT elongation and risk of adrenal insufficiency could limit the feasibility of this approach. Moreover, osilodrostat did not exert a specific effect on tumor volume thus cannot replace the indication of pasireotide to stabilize or reduce adenoma size (9).

Biochemical control of hypercortisolism with cortisol-lowering drugs is expected to be less effective on comorbidities than surgical remission (46). Nevertheless, apart from glucose homeostasis, pasireotide previously proved beneficial on blood pressure, lipid profile, and body weight (47). In our study, in addition to changes in SBP and DBP, we also evaluated the number and dosage of antihypertensive therapies (see **Supplementary Table 1**) and confirmed pasireotide's favorable effect on pressure profile, especially in the long term. This finding was evident despite the association with metyrapone in seven cases, which might cause hypertension due to the accumulation of mineralocorticoid precursors (48). Even after excluding patients on anorectic drugs, a significant reduction in BMI was observed and an add-on treatment seemed to provide further benefit. Apart from treatment effect, the role of lifestyle modification aimed at diabetes risk reduction cannot be ruled out. Regarding dyslipidemia, we found a high prevalence in our cohort (89%), even though some of the cases were already receiving statin treatment. This was consistent with active CD being considered at high cardiovascular risk according to the most recent consensus (1). Notably, the association of metabolic and CV comorbidities or the complications (e.g., acute coronary syndrome) may suffice to label patients with CD as having high or very high CV risk also based on the criteria used in the general population (20). Although a trend to LDLc reduction was observed while on cortisol-lowering treatments (**Table 4**), achieving ambitious lipid targets likely requires the use of lipid-lowering drugs. While recommending statin treatment in most patients with CD seems reasonable, the lack of disease-specific studies should be acknowledged, as current recommendations rely on guidelines developed for the general population (1).

Our study has several limitations, the most relevant being the small sample size especially regarding combination treatment (i.e., only seven cases received pasireotide plus metyrapone). Because of its retrospective design, few data were missing. Moreover, only one patient was affected by a severe disease, while all the others presented a mild to moderate form; thus, our considerations are inherent to these cases. The clinical judgment about the time and the choice of the add-on treatment was based on the physician’s experience. Of note, pasireotide was not increased to the maximum dosage in many patients due to tolerability issues (i.e., only three patients received 900 μg BID); also, cabergoline and metyrapone dosages could still be up-titrated in some patients. Blood pressure profiles were assessed during ambulatory visits, without the use of a 24-h monitoring device. There was also heterogeneity in natural history since we included both *de novo* cases and those who underwent multiple treatments, including pituitary irradiation that may have contributed to biochemical control in a subset of our patients. Still, this latter bias seems limited as despite the larger use of combination treatment, previously irradiated cases showed similar reductions of UFC and LNSC (from baseline to the last follow-up) to non-irradiated cases (**Supplementary Table 2**); four out five cases were irradiated at least 15 years prior to the start of pasireotide treatment. Furthermore, patients who have received pituitary irradiation often face limitations in enrolling in clinical studies; therefore, data on this population require support from real-world evidence.

Our experience confirms the efficacy of pasireotide in controlling UFC, especially in combined regimens, as well as the difficulty of restoring circadian rhythm in CD, which may require higher doses and prolonged treatments. We report for the first time the association of pasireotide with metyrapone. This approach appears useful especially in cases with poor tolerance or hyperglycemia, but larger studies are needed to better assess the effectiveness of this approach. While pasireotide adversely affects glucose homeostasis, its positive effects, either alone or in combination, on blood pressure, lipid profile, and body weight justify its use under careful hyperglycemia management.

## Data Availability

The raw data supporting the conclusions of this article will be made available by the authors, without undue reservation.

## References

[1] Fleseriu M Auchus R Bancos I Ben-Shlomo A Bertherat J Biermasz NR . Consensus on diagnosis and management of Cushing’s disease: a guideline update. Lancet Diabetes Endocrinol. (2021) 9:847–75. doi: 10.1016/S2213-8587(21)00235-7, PMID: 34687601 PMC8743006

[2] Lacroix A Gu F Gallardo W Pivonello R Yu Y Witek P . Efficacy and safety of once-monthly pasireotide in Cushing’s disease: a 12 month clinical trial. Lancet Diabetes Endocrinol. (2018) 6:17–26. doi: 10.1016/S2213-8587(17)30326-1, PMID: 29032078

[3] Gadelha M Bex M Feelders RA Heaney AP Auchus RJ Gilis-Januszewska A . Randomized trial of osilodrostat for the treatment of cushing disease. J Clin Endocrinol Metab. (2022) 107:e2882–95. doi: 10.1210/clinem/dgac178, PMID: 35325149 PMC9202723

[4] Pivonello R Bancos I Feelders RA Kargi AY Kerr JM Gordon MB . Relacorilant, a selective glucocorticoid receptor modulator, induces clinical improvements in patients with cushing syndrome: results from A prospective, open-label phase 2 study. Front Endocrinol (Lausanne). (2021) 12. doi: 10.3389/fendo.2021.662865, PMID: 34335465 PMC8317576

[5] Findling JW Fleseriu M Newell-Price J Petersenn S Pivonello R Kandra A . Late-night salivary cortisol may be valuable for assessing treatment response in patients with Cushing’s disease: 12-month, Phase III pasireotide study. Endocrine. (2016) 54:516–23. doi: 10.1007/s12020-016-0978-6, PMID: 27209465 PMC5083774

[6] Newell-Price J Fleseriu M Pivonello R Feelders RA Gadelha MR Lacroix A . Improved clinical outcomes during long-term osilodrostat treatment of cushing disease with normalization of late-night salivary cortisol and urinary free cortisol. J Endocr Soc. (2024) 9. doi: 10.1210/jendso/bvae201, PMID: 39610378 PMC11604051

[7] de Bruin C Feelders RA Waaijers AM van Koetsveld PM Sprij-Mooij DM Lamberts SWJ . Differential regulation of human dopamine D2 and somatostatin receptor subtype expression by glucocorticoids *in vitro*. J Mol Endocrinol. (2009) 42:47–56. doi: 10.1677/JME-08-0110, PMID: 18852217

[8] Petersenn S Salgado LR Schopohl J Portocarrero-Ortiz L Arnaldi G Lacroix A . Long-term treatment of Cushing’s disease with pasireotide: 5-year results from an open-label extension study of a Phase III trial. Endocrine. (2017) 57:156–65. doi: 10.1007/s12020-017-1316-3, PMID: 28597198 PMC5486525

[9] Mondin A Manara R Voltan G Tizianel I Denaro L Ferrari M . Pasireotide-induced shrinkage in GH and ACTH secreting pituitary adenoma: A systematic review and meta-analysis. Front Endocrinol (Lausanne). (2022) 13. doi: 10.3389/fendo.2022.935759, PMID: 35846311 PMC9283714

[10] Feelders RA de Bruin C Pereira AM Romijn JA Netea-Maier RT Hermus AR . Pasireotide alone or with cabergoline and ketoconazole in cushing’s disease. New Engl J Med. (2010) 362:1846–8. doi: 10.1056/NEJMc1000094, PMID: 20463350

[11] Feelders RA Fleseriu M Kadioglu P Bex M González-Devia D Boguszewski CL . Long-term efficacy and safety of subcutaneous pasireotide alone or in combination with cabergoline in Cushing’s disease. Front Endocrinol (Lausanne). (2023) 14. doi: 10.3389/fendo.2023.1165681, PMID: 37876540 PMC10593462

[12] Barbot M Guarnotta V Zilio M Ceccato F Ciresi A Daniele A . Effects of pasireotide treatment on coagulative profile: a prospective study in patients with Cushing’s disease. Endocrine. (2018) 62:207–14. doi: 10.1007/s12020-018-1669-2, PMID: 29980915

[13] Barbot M Regazzo D Mondin A Zilio M Lizzul L Zaninotto M . Is pasireotide-induced diabetes mellitus predictable? A pilot study on the effect of a single dose of pasireotide on glucose homeostasis. Pituitary. (2020) 23:534–42. doi: 10.1007/s11102-020-01055-x, PMID: 32524277

[14] Barbot M Mondin A Regazzo D Guarnotta V Basso D Giordano C . Incretin response to mixed meal challenge in active cushing’s disease and after pasireotide therapy. Int J Mol Sci. (2022) 23:5217. doi: 10.3390/ijms23095217, PMID: 35563608 PMC9105040

[15] Ceccato F Barbot M Mondin A Boscaro M Fleseriu M Scaroni C . Dynamic testing for differential diagnosis of ACTH-dependent cushing syndrome: A systematic review and meta-analysis. J Clin Endocrinol Metab. (2023) 108:e178–88. doi: 10.1210/clinem/dgac686, PMID: 36453141

[16] Petersenn S Newell-Price J Findling JW Gu F Maldonado M Sen K . High variability in baseline urinary free cortisol values in patients with Cushing’s disease. Clin Endocrinol (Oxf). (2014) 80:261–9. doi: 10.1111/cen.12259, PMID: 23746264 PMC4231220

[17] Sandouk Z Johnston P Bunch D Wang S Bena J Hamrahian A . Variability of late-night salivary cortisol in cushing disease: A prospective study. J Clin Endocrinol Metab. (2018) 103:983–90. doi: 10.1210/jc.2017-02020, PMID: 29329418

[18] McEvoy JW McCarthy CP Bruno RM Brouwers S Canavan MD Ceconi C . 2024 ESC Guidelines for the management of elevated blood pressure and hypertension. Eur Heart J. (2024) 45:3912–4018. doi: 10.1093/eurheartj/ehae178, PMID: 39210715

[19] American Diabetes Association Professional Practice Committee . Classification and diagnosis of diabetes: standards of medical care in diabetes-2022. Diabetes Care. (2022) 45:S17–38. doi: 10.2337/dc22-S002, PMID: 34964875

[20] Mach F Baigent C Catapano AL Koskinas KC Casula M Badimon L . 2019 ESC/EAS Guidelines for the management of dyslipidaemias: lipid modification to reduce cardiovascular risk. Eur Heart J. (2020) 41:111–88. doi: 10.1093/eurheartj/ehz455, PMID: 31504418

[21] Yumuk V Tsigos C Fried M Schindler K Busetto L Micic D . European guidelines for obesity management in adults. Obes Facts. (2015) 8:402–24. doi: 10.1159/000442721, PMID: 26641646 PMC5644856

[22] Albani A Ferraù F Ciresi A Pivonello R Scaroni C Iacuaniello D . Pasireotide treatment reduces cardiometabolic risk in Cushing’s disease patients: an Italian, multicenter study. Endocrine. (2018) 61:118–24. doi: 10.1007/s12020-018-1524-5, PMID: 29383677

[23] Vilar L Naves LA Azevedo MF Arruda MJ Arahata CM Moura e Silva L . Effectiveness of cabergoline in monotherapy and combined with ketoconazole in the management of Cushing’s disease. Pituitary. (2010) 13:123–9. doi: 10.1007/s11102-009-0209-8, PMID: 19943118

[24] Kamenický P Droumaguet C Salenave S Blanchard A Jublanc C Gautier JF . Mitotane, metyrapone, and ketoconazole combination therapy as an alternative to rescue adrenalectomy for severe ACTH-dependent cushing’s syndrome. J Clin Endocrinol Metab. (2011) 96:2796–804. doi: 10.1210/jc.2011-0536, PMID: 21752886

[25] Valassi E Crespo I Gich I Rodríguez J Webb SM . A reappraisal of the medical therapy with steroidogenesis inhibitors in C ushing’s syndrome. Clin Endocrinol (Oxf). (2012) 77:735–42. doi: 10.1111/j.1365-2265.2012.04424.x, PMID: 22533782

[26] Barbot M Albiger N Ceccato F Zilio M Frigo AC Denaro L . Combination therapy for Cushing’s disease: effectiveness of two schedules of treatment. Should we start with cabergoline or ketoconazole? Pituitary. (2014) 17:109–17. doi: 10.1007/s11102-013-0475-3, PMID: 23468128

[27] Corcuff JB Young J Masquefa-Giraud P Chanson P Baudin E Tabarin A . Rapid control of severe neoplastic hypercortisolism with metyrapone and ketoconazole. Eur J Endocrinol. (2015) 172:473–81. doi: 10.1530/EJE-14-0913, PMID: 25624013

[28] Dormoy A Haissaguerre M Vitellius G Do Cao C Geslot A Drui D . Efficacy and safety of osilodrostat in paraneoplastic cushing syndrome: A real-world multicenter study in France. J Clin Endocrinol Metab. (2023) 108:1475–87. doi: 10.1210/clinem/dgac691, PMID: 36470583 PMC10188310

[29] Paes T van der Pas R van Koetsveld PM Dogan F van den Berge KKA Netea-Maier RT . A prospective trial with ketoconazole induction therapy and octreotide maintenance treatment for cushing’s disease. J Endocr Soc. (2025) 9. doi: 10.1210/jendso/bvaf089, PMID: 40452800 PMC12123513

[30] Vilar L Naves LA MaChado MC Bronstein MD . Medical combination therapies in Cushing’s disease. Pituitary. (2015) 18:253–62. doi: 10.1007/s11102-015-0641-x, PMID: 25647330

[31] Gilis-Januszewska A Bogusławska A Rzepka E Ziaja W Hubalewska-Dydejczyk A . Individualized medical treatment options in Cushing disease. Front Endocrinol (Lausanne). (2022) 13. doi: 10.3389/fendo.2022.1060884, PMID: 36531477 PMC9755355

[32] Regazzo D Mondin A Scaroni C Occhi G Barbot M . The role of glucocorticoid receptor in the pathophysiology of pituitary corticotroph adenomas. Int J Mol Sci. (2022) 23:6469. doi: 10.3390/ijms23126469, PMID: 35742910 PMC9224504

[33] Nieman LK Biller BMK Findling JW Newell-Price J Savage MO Stewart PM . The diagnosis of cushing’s syndrome: an endocrine society clinical practice guideline. J Clin Endocrinol Metab. (2008) 93:1526–40. doi: 10.1210/jc.2008-0125, PMID: 18334580 PMC2386281

[34] Newell-Price J Pivonello R Tabarin A Fleseriu M Witek P Gadelha MR . Use of late-night salivary cortisol to monitor response to medical treatment in Cushing’s disease. Eur J Endocrinol. (2020) 182:207–17. doi: 10.1530/EJE-19-0695, PMID: 31804965 PMC7003692

[35] Minnetti M Hasenmajer V Pofi R Venneri MA Alexandraki KI Isidori AM . Fixing the broken clock in adrenal disorders: focus on glucocorticoids and chronotherapy. J Endocrinol. (2020) 246:R13–31. doi: 10.1530/JOE-20-0066, PMID: 32380472

[36] Eguchi K Kawamoto K Uozumi T Ito A Arita K Kurisu K . Effect of cabergoline, a dopamine agonist, on estrogen-induced rat pituitary tumors: *in vitro* culture studies. Endocr J. (1995) 42:413–20. doi: 10.1507/endocrj.42.413, PMID: 7670571

[37] Eguchi K Kawamoto K Uozumi T Ito A Arita K Kurisu K . *In vivo* effect of cabergoline, a dopamine agonist, on estrogen-induced rat pituitary tumors. Endocr J. (1995) 42:153–61. doi: 10.1507/endocrj.42.153, PMID: 7627259

[38] Lin SJ Leng ZG Guo YH Cai L Cai Y Li N . Suppression of mTOR pathway and induction of autophagy-dependent cell death by cabergoline. Oncotarget. (2015) 6:39329–41. doi: 10.18632/oncotarget.5744, PMID: 26513171 10.18632/oncotarget.5744 PMC4770775

[39] Batista DL Zhang X Gejman R Ansell PJ Zhou Y Johnson SA . The effects of SOM230 on cell proliferation and adrenocorticotropin secretion in human corticotroph pituitary adenomas. J Clin Endocrinol Metab. (2006) 91:4482–8. doi: 10.1210/jc.2006-1245, PMID: 16940446

[40] Murasawa S Kageyama K Sugiyama A Ishigame N Niioka K Suda T . Inhibitory effects of SOM230 on adrenocorticotropic hormone production and corticotroph tumor cell proliferation *in vitro* and in *vivo*. Mol Cell Endocrinol. (2014) 394:37–46. doi: 10.1016/j.mce.2014.07.001, PMID: 25011056

[41] Zatelli MC Piccin D Vignali C Tagliati F Ambrosio MR Bondanelli M . Pasireotide, a multiple somatostatin receptor subtypes ligand, reduces cell viability in non-functioning pituitary adenomas by inhibiting vascular endothelial growth factor secretion. Endocr Relat Cancer. (2007) 14:91–102. doi: 10.1677/ERC-06-0026, PMID: 17395978

[42] Samson SL Gu F Feldt-Rasmussen U Zhang S Yu Y Witek P . Managing pasireotide-associated hyperglycemia: a randomized, open-label, Phase IV study. Pituitary. (2021) 24:887–903. doi: 10.1007/s11102-021-01161-4, PMID: 34275099 PMC8550309

[43] Henry RR Ciaraldi TP Armstrong D Burke P Ligueros-Saylan M Mudaliar S . Hyperglycemia associated with pasireotide: results from a mechanistic study in healthy volunteers. J Clin Endocrinol Metab. (2013) 98:3446–53. doi: 10.1210/jc.2013-1771, PMID: 23733372

[44] Fleseriu M Petersenn S Biller BMK Kadioglu P De Block C T’Sjoen G . Long-term efficacy and safety of once-monthly pasireotide in Cushing’s disease: A Phase III extension study. Clin Endocrinol (Oxf). (2019) 91:776–85. doi: 10.1111/cen.14081, PMID: 31465533 PMC6899900

[45] Pivonello R Simeoli C Di Paola N Larocca A Crescenzo EM Colao A . Osilodrostat: A novel potent inhibitor of 11-beta-hydroxylase for the treatment of cushing’s syndrome. touchREVIEWS. Endocrinology. (2024) 20. doi: 10.17925/EE.2024.20.1.8, PMID: 38812665 PMC11132648

[46] Tizianel I Lizzul L Mondin A Voltan G Mazzeo P Scaroni C . Cardiometabolic complications after Cushing’s disease remission. J Endocrinol Invest. (2025) 48, 1597–1610. doi: 10.1007/s40618-025-02572-x, PMID: 40138148 PMC12313713

[47] Pivonello R Petersenn S Newell-Price J Findling JW Gu F Maldonado M . Pasireotide treatment significantly improves clinical signs and symptoms in patients with Cushing’s disease: results from a Phase III study. Clin Endocrinol (Oxf). (2014) 81:408–17. doi: 10.1111/cen.12431, PMID: 24533697

[48] Fallo F Di Dalmazi G Beuschlein F Biermasz NR Castinetti F Elenkova A . Diagnosis and management of hypertension in patients with Cushing’s syndrome: a position statement and consensus of the Working Group on Endocrine Hypertension of the European Society of Hypertension. J Hypertens. (2022) 40:2085–101. doi: 10.1097/HJH.0000000000003252, PMID: 35950979

