# Supplementary material for: Real-life data on pasireotide in monotherapy or combined in active Cushing’s disease
Source: Front Endocrinol (Lausanne). 2025 Nov 13;16:1695342. doi: 10.3389/fendo.2025.1695342 (PMC12657153; doi:10.3389/fendo.2025.1695342)
Supplement: Supplementary file 1 [file DataSheet1.pdf]

Real-life data on pasireotide in monotherapy or combined in active Cushing's Disease

Alessandro Mondin[1,2], Filippo Ceccato[1,2], Carla Scaroni[1,2], Luca Denaro[3], Renzo Manara[4,5], Umberto Maria Robertazzo[4,5], Mattia Barbot[1,2]

[1] *Department of Medicine-DIMED, University of Padova, Padova, Italy.*

[2] *Endocrinology Unit, University Hospital of Padova, Padova, Italy*

[3] *Academic Neurosurgery, Department of Neurosciences, University of Padova, Padova, Italy.*

[4] *Department of Neuroscience (DNS), University of Padova, Padova, Italy.*

[5] *Neuroradiology Unit, University-Hospital of Padova, Padova, Italy.*

Supplementary data

Supplementary Figure 1. *Main reasons for pasireotide (alone or combined) discontinuation. * Well tolerated treatment, switch to another drug due to reduced compliance (patient unwilling to continue with daily subcutaneous treatment) and/or failure to achieve biochemical control. ** 1st surgery in 3 cases.*

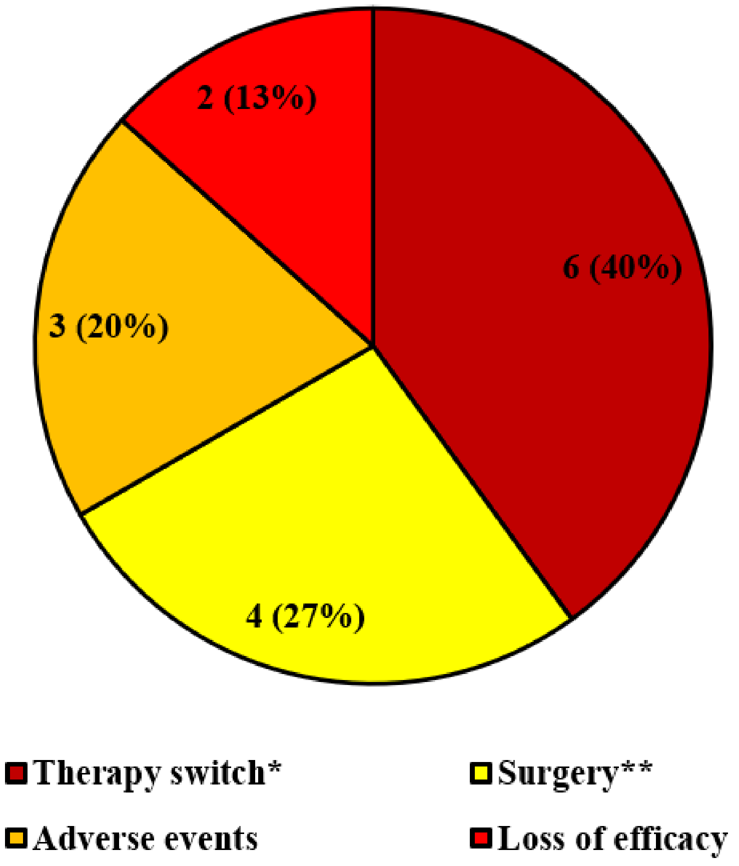

Supplementary Table 1. *Pressure profile following pasireotide (alone or in combination) treatment. SBP: systolic blood pressure; DBP: diastolic blood pressure.*

	**Improvement**	**No changes**	**Worsening**
**Criteria**	a. Antihypertensive dose/ number reduction with stable BP (ΔSBP<10 mmHg, ΔDBP<5 mmHg).<br><br>b. Stable antihypertensive therapy with SBP decrease ≥10 mmHg and/or DBP decrease ≥5 mmHg.	a. Stable antihypertensive therapy with ΔSBP<10 mmHg and ΔDBP<5 mmHg.<br><br>b. Antihypertensive dose/ number increase accompanied by a decrease ≥ 10 mmHg in SBP and ≥5 mmHg in DBP.<br><br>c. Antihypertensive dose/ number decrease accompanied by an increase ≥ 10 mmHg in SBP and ≥5 mmHg in DBP.	a. Antihypertensive dose/ number increase with stable BP (ΔSBP<10 mmHg, ΔDBP<5 mmHg).<br><br>b. Stable antihypertensive therapy with SBP increase ≥10 mmHg and/or DBP increase ≥5 mmHg.
**Overall**	9 (53%)	5 (29%)	3 (18%)
**Over 1 year of follow up**	5 (50%)	5 (50%)	0 (0%)

Supplementary Table 2. *Reduction of UFC or LNSC urinary free cortisol (UFC) and late-night salivary cortisol (LNSC) from baseline to the last follow up in patients divided by pituitary irradiation. RT: radiotherapy; N: number; ULN: upper limit of normal.*

	**UFC decrease**		**LNSC decrease**	
**RT**	yes	no	yes	no
**N**	4	12	2	10
**Value (n-fold ULN)**	1.0	1.3	1.0	0.56
**p**	0.77		0.61	
